# Parvovirus B19 infection as a trigger of acute cholestasis in heterozygous genetic BSEP deficiency: a case report and review of the literature of acute parvovirus B19-related hepatitis

**DOI:** 10.1007/s11739-026-04464-1

**Published:** 2026-07-24

**Authors:** Madalina-Gabriela Indre, Giovanni Vitale, Amalia Conti, Maria Boe, Deborah Malvi, Simona Ferrari, Mariarosaria Marseglia, Liliana Gabrielli, Tiziana Lazzarotto, Fabio Piscaglia

**Affiliations:** 1 Department of Medical and Surgical Sciences, University of Bologna, 40138 Bologna, Italy; 2 Division of Internal Medicine, Hepatobiliary and Immunoallergic Diseases, IRCCS Azienda Ospedaliero-Universitaria Di Bologna, Bologna, Italy; 3 Internal Medicine Unit for the Treatment of Severe Organ Failure, IRCCS AOUBO, 40138 Bologna, Italy; 4 Medical Genetics Unit, IRCCS, Azienda Ospedaliero-Universitaria Di Bologna, 40138 Bologna, Italy; 5 Pathology Unit, IRCCS Azienda Ospedaliero-Universitaria Di Bologna, 40138 Bologna, Italy; 6 Unit of Microbiology, IRCCS Azienda Ospedaliero-Universitaria Di Bologna, 40138 Bologna, Italy

**Keywords:** BSEP deficiency, ABCB11, Cholestasis, Hepatitis, Parvovirus B19

## Abstract

Bile salt export pump (BSEP) deficiency, also termed progressive familial intrahepatic cholestasis type 2 (PFIC2), is a rare genetic disorder caused by mutations in the ATP-binding cassette, sub-family B member 11 (ABCB11) gene, predisposing to cholestasis and progressive liver damage. Although parvovirus B19 is a recognized but rare cause of acute hepatitis in children, its role in adult liver disease remains uncertain. To expand the knowledge on this topic, we performed a narrative review of published adult cases of B19-associated hepatitis and report the case of a 46-year-old immunocompetent man who developed acute cholestatic hepatitis during B19 infection. The clinical course was not fully attributable to the viral insult alone, prompting further evaluation. Liver histology demonstrated marked loss of BSEP immunoreactivity, and genetic testing identified a heterozygous ABCB11 variant. Despite treatment with ursodeoxycholic acid and cholestyramine, the symptoms persisted, leading to the introduction of the ileal bile acid transporter (IBAT) inhibitor, odevixibat, which resulted in rapid relief of pruritus and near-complete biochemical normalization within 2 months. Our review identified 32 published adult cases of B19-associated hepatitis. Most patients were immunocompetent, with presentations ranging from mild hepatic dysfunction to fulminant liver failure requiring transplantation. In more than half of the patients, predisposing factors such as hematologic disorders or viral co-infections were identified. This review highlights the importance of considering parvovirus B19 infection in the differential diagnosis of acute hepatitis in adults and of investigating potential underlying predisposing factors when the clinical course cannot be fully explained by the viral infection alone.

**Supplementary Information:**

The online version contains supplementary material available at https://doi.org/10.1007/s11739-026-04464-1.

## Introduction

Parvovirus B19 (B19) was first linked to clinical disease in 1981 [1]. While traditionally considered a rare cause of acute hepatitis in children and infants, its role in adult liver disease, particularly acute hepatitis and fulminant hepatic failure, remains controversial [2, 3]. Nevertheless, increasing evidence supports its potential to induce liver dysfunction in adults, especially in those with underlying susceptibility [4]. To further expand the current knowledge on B19-associated hepatitis in adults, we conducted a narrative review of published cases. Here, we also report the case of an adult patient who developed acute cholestatic hepatitis associated with B19 infection in the setting of a previously unrecognized BSEP deficiency. The interaction between the underlying genetic predisposition and the viral infection likely contributed to a more severe clinical course. The host-related factors may influence the hepatic manifestations and severity of B19 infection; therefore, a better understanding of underlying susceptibility conditions is essential for interpreting the wide spectrum of clinical presentations observed in affected patients.

Bile salt export pump (BSEP) deficiency, or progressive familial intrahepatic cholestasis type 2 (PFIC2), is a genetic liver disorder caused by mutations in the ATP-binding cassette, sub-family B member 11 (ABCB11) gene [5] that belongs to the group of low gamma-glutamyl transferase (GGT) cholestatic diseases [6]. The condition disrupts bile flow at the hepatocellular level and predisposes patients to cholestasis and progressive liver injury. This may ultimately lead to liver transplantation, especially in a homozygous state, making it a leading cause of pediatric liver transplantation [5]. Conversely, in adolescents and adults, a heterozygous variant in PFIC-related genes is associated with variable phenotypes, ranging from acute or chronic forms of liver disease, progressive or non-progressive, such as intrahepatic cholestasis of pregnancy, drug-induced cholestasis, low phospholipid-associated cholelithiasis, recurrent intrahepatic cholestasis, and chronic idiopathic cholestasis [7]. In this type of vulnerable hepatic environment, additional factors, such as viral infections, can precipitate or exacerbate liver dysfunction, potentially leading to overt clinical manifestations. Considering this, the identification of underlying susceptibility factors becomes particularly relevant in patients presenting with unexplained liver injury.

This review also highlights the growing role of precision medicine in hepatology. The use of advanced diagnostic tools, including genetic testing, enabled the identification of an underlying susceptibility factor that might have amplified the hepatic manifestations of B19 infection. The recognition of this genetic background facilitated accurate diagnosis, aided in individualized management, and ultimately contributed to a favorable clinical outcome.

## Methods

This narrative review summarizes the currently available evidence regarding acute hepatitis associated with parvovirus B19 infection in immunocompetent adults. A structured literature search was conducted in PubMed/MEDLINE database up to February 2025. The search strategy comprised combinations of the following keywords: “parvovirus B19”, “human parvovirus B19”, “acute hepatitis”, “cholestatic hepatitis”, “liver injury”, “hepatitis”, and “cholestasis”.

The articles published in English reporting adult cases of parvovirus B19 infection-associated hepatitis were considered. Studies involving pediatric patients and non-human studies were excluded. The identified cases were reviewed descriptively, and relevant demographic, clinical, laboratory, histological, and outcome data were extracted and summarized. No formal PRISMA-based systematic methodology or quantitative meta-analysis was performed, as the objective of this work was to provide a narrative, clinically oriented synthesis of available evidence rather than a pooled quantitative estimate.

In addition, we reported an illustrative clinical case of B19-associated acute cholestatic hepatitis occurring in an immunocompetent adult patient with a heterozygous pathogenic ABCB11 variant resulting in partial BSEP deficiency.

### Pathogenesis of parvovirus B19 infection

B19 is a member of the Parvoviridae family, subfamily Parvovirinae genus, Erythrovirus [8]. It is the only known human-pathogenic virus within this family. B19 was first identified in 1975 during the screening of asymptomatic individuals for hepatitis B virus (HBV) infection [9]). It is a widespread virus that can be associated with a range of different pathologies and clinical manifestations [10, 11]. The clinical spectrum of B19 infection continues to broaden, as increasing numbers of case reports and clinical studies are published. Most of the B19 infections occur during childhood [1], with approximately 50% of the individuals seropositive by age 18–19 [12, 13], and up to 65% of adults showing evidence of prior exposure [14]. Structurally, B19 is a small, non-enveloped virus with a single-stranded deoxyribonucleic acid (DNA) genome approximately 5000 nucleotides in length. This genome encodes two structural capsid proteins (VP1 and VP2) and a single non-structural protein (NS1), essential for viral replication and pathogenesis [15, 16]. VP1 has an additional 227 aminoacids at the N-terminus, a unique region referred to as VP1u, which plays a critical role in viral infectivity and immune recognition [17]. The infection is transmitted by respiratory droplets, blood products, stem cells or solid organ transplantation [18–22], vertical transmission or the placental route [23]. Nosocomial transmission has also been documented and patients with persistent infection seropositivity should be regarded as potentially infectious [24]. B19 exhibits a marked tropism for erythroid progenitor cells in the bone marrow [10, 25–27]. It binds specifically to the neutral glycosphingolipid globoside (Gb4), also known as the blood group P antigen, which is abundantly expressed on the surface of erythroid precursors. This binding leads to the externalization of the VP1u, a critical step for viral entry [28]. A wide distribution of B19 receptors in human erythroid and non-erythroid tissues, such as hepatocytes, endothelial cells (vascular and intracardiac), placental trophoblastic cells, and some megakaryocytes cells, might explain the ability of B19 to cause multiorgan diseases [29]. Integrins and other surface molecules facilitate internalization [30, 31]. It is presumed that this primary interaction may be responsible for the systemic dissemination of the virus [30]. Parvovirus might also induce the production of autoantibodies [32].

The mechanism by which B19 infects and damages hepatocytes remains incompletely understood. Basic research has demonstrated that B19 is capable of infecting and inducing apoptosis in both primary hepatocytes and liver-derived cell lines [33]. A direct cytopathic effect has been proposed [34], in which the non-structural protein NS1 plays a central role [35]. NS1 functions as a cytotoxic protein capable of inducing IL-6 production and promoting apoptosis [36]. NS1 expression has also been shown to activate both caspase-9 and caspase-3, enzymes essential for the execution of apoptosis [33, 35]. In HepG2 cells, apoptosis might be triggered, therefore, by internal stress signals within the infected cell, rather than by external activation of tumor necrosis factor (TNF) receptor family members [33]. An indirect, immunologically mediated mechanism has also been proposed [37]. B19 can trigger immune responses that lead to the release of proinflammatory cytokines such as interferon-gamma (IFN-γ) and tumor necrosis factor-alpha (TNF-α), which may contribute to liver cell injury [38, 39].

### Markers of parvovirus B19 infection in adults

Diagnosis of B19 infection primarily relies on the detection of virus-specific IgM and IgG antibodies. Detectable B19 DNA typically develops approximately 5–6 days after infection [38, 39]. IgM is the first antibody to appear—usually within 10–14 days post-infection—and serves as a marker of recent or acute infection [9] and remains detectable for up to 2–3 months [9]. The IgM response is usually followed by production of IgG [9] that indicates immunity for B19 infection and these antibodies are typically detected lifelong [39]. B19 specific IgG antibodies are considered to protect from reinfection, however, there are reports suggesting that the reactivation of the virus is possible [40]. Viral DNA can typically be detected in serum for up to 6 months after symptom onset [41]. In addition to serological and molecular diagnostics, the detection of VP1- and VP2-specific CD4 + T cells, particularly VP1u-specific subsets, may offer further insight into the infection status, as these are sometimes found in patients with acute or persistent infection [42].

The detection of parvovirus B19 DNA in a liver biopsy specimen, without a consistent clinical picture, does not, on its own, necessarily indicate that the patient is suffering from an acute infection with B19 [43].

The studies have shown that B19 infection is more prevalent in cancer patients compared to healthy individuals, suggesting an association between immunocompromised states and enhanced viral persistence or reactivation [44, 45]. During periods of immunosuppression or co-infection with other pathogens, B19 viral load may increase significantly [16]. Some experts suggested that B19 might act as a cofactor for liver damage alongside other hepatotoxic agents, such as paracetamol [46].

### Initial presentation and extrahepatic symptoms

Asymptomatic infection has been reported to occur in about one-quarter of adults [47]. Depending upon the age, condition of the immune system, and hematologic comorbidities, different people have varying clinical presentations. B19 infection is generally self-healing in immunocompetent individuals, but it can cause fatal effects in high-risk groups [48]. The wide distribution of B19 receptors in human erythroid and non-erythroid tissues, such as hepatocytes, endothelial cells (vascular and intracardiac), placental trophoblastic cells, and some megakaryocytes cells, explain the ability of B19 to cause multiorgan diseases [29]. Most of the B19 infections that are not asymptomatic present symptoms similar to a ‘common cold’ [49, 50]. In early infection, non-specific flu-like symptoms are present, due to viral replication in the upper respiratory tract [16, 18]. From the respiratory epithelium, the virus particles access the bloodstream by an unknown mechanism [15], reaching the bone marrow. This causes fever and nonspecific, flu-like symptoms [51]. The virus has a marked tropism for erythroid precursors in the bone marrow, leading to possible hematological abnormalities such as aplastic anemia and hemolytic anemia [28, 52–55]. B19 can cause severe, often fatal, hematopoietic damage, as evidenced by four cases of hemophagocytic syndrome [56], one case of bone marrow necrosis [57], one case of long-lasting red cell aplasia [11]. On rare occasions, it can mimic leukemic or myelodysplastic conditions [58, 59]. It can cause neutropenia and thrombocytopenia, but they are less commonly observed [60].As the infection continues,, IgM antibodies develop and bind to the virus forming circulating immune complexes, which are responsible for the skin and articular extrahepatic manifestations [51]. Many patients display maculopapular exanthema, erythema infectiosum (fifth disease) [4, 61], arthralgia or poly-arthropathy often with rheumatoid-like involvement, or systemic lupus erythematosus, particularly in middle aged women [1, 62, 63]. In adults, the skin eruption can be transient with less specific features, sparing the face [47]. In adult patients with underlying immunodeficiency, B19 may cause chronic disease [10, 11], or possible chronic bone marrow failure [1].

B19 infection has also been reported to affect the respiratory system and cause interstitial lung disease, severe pneumonia, and acute respiratory distress syndrome (ARDS), kidney diseases (acute glomerulonephritis and nephropathy syndrome), heart diseases such as myocarditis or heart failure (HF), particularly in patients with abnormal hematological parameters or immune status [2, 4, 18, 25, 64–69]. Neurological and cardiac manifestations are rare [63, 70].

### Hepatic involvement in parvovirus B19 infection

B19 has been identified as an uncommon but potential cause of acute hepatitis, particularly in children and infants [2, 3]. Increased levels of liver enzymes have been observed in neonates surviving B19 infection in utero [71]. However, its etiological role in acute liver injury remains a subject of ongoing debate, especially in adult patients [72, 73]. This uncertainty largely stems from the frequent detection of B19 DNA in various tissues, including hepatic tissue, even in the absence of clinically significant liver disease [73, 74], or long after the resolution of the acute hepatitis [75]. There is also insufficient evidence to clarify whether the hepatic inflammation is due to direct viral effects or immune-mediated mechanisms [76]. As viral DNA can be detected in liver tissue, it has been assumed that the liver might represent the target organ for persistent B19 infection in adults [42, 73, 77]. Evidence in favor of this association continues to accumulate. In a study by Langnas et al., B19 DNA was detected in liver tissue obtained from 4 of 6 patients with fulminant hepatitis-associated aplastic anemia, from 2 of 4 patients with cryptogenic fulminant hepatitis, and from 5 of 34 patients with chronic liver disease [10]. Despite these observations, some other clinical studies have reported conflicting findings. One study found no clear association between B19 and acute liver failure; nevertheless, the possibility that the virus might have contributed to transient elevations in liver enzymes has not been excluded [78].

B19 has been claimed to act as a co-infectant in chronic hepatitis B and C virus infections, often predicting worse outcomes [76, 79, 80]. Other reports suggested the virus to be the cause of acute non- A–E hepatitis and liver failure [37, 80, 81]. Additionally, it has been reported as a primary cause of hepatitis in several case reports, which are summarized in this narrative review.

### Evidence from published cases of parvovirus B19-associated hepatitis in adults

We identified 32 case reports [18, 40, 42, 43, 46, 48, 68, 72, 82–100] in the literature describing B19-associated hepatitis in adult patients, as detailed in Table 1. These cases highlight the diverse clinical presentations and underline the need for increased awareness about B19 as a potential cause of acute liver injury in adults. The cases originated from various geographical regions, including Europe (15 cases, 46.9%) [42, 43, 46, 68, 72, 83, 84, 86, 91, 96, 97, 99], Asia (9 cases, 28.1%) [40, 48, 85, 88, 90, 93–95], and the Americas (8 cases, 25%) [18, 82, 87, 89, 92, 98, 100]. Patient ages ranged from 18 to 58 years. The majority were female, with 21 cases (65.6%) reported in women and 11 (34.4%) in men. Notably, most patients, 28 out of 32 (87%), were reported as immunocompetent, indicating that the condition can occur even in the absence of overt immunosuppression. Among the four immunocompromised individuals, the first was undergoing chemotherapy [40], the second was under pegylated interferon alpha 2a treatment for chronic HBV infection [83], the third had HIV [96], and the fourth had moderate idiopathic lymphopenia [91].

**Table 1:** Documented cases of parvovirus B19-associated hepatitis in adults

No	Author	Year	Country	Biological sex	Age (years)	Immune status	Liver disease	Particularity of the case	Outcome	Time to resolution/Other
1	Khan [82]	2022	USA	Female	43	Immunocompetent	Acute hepatitis	B19-induced hepatitis with hepatosplenomegaly and seronegative symmetrical polyarthropathy	Resolution	N/R
2	Luo J [48]	2021	China	Male	43	Immunocompetent	Acute BV19 on chronic AH	Disseminated B19 infection-induced MODS in patient with AH. Self-limiting B19 infections in seven nurses caring for the patient expressed as self-limiting erythema infectiosum	Death	Death
3	Gille N [46]	2020	France	Male	18	Immunocompetent	Fulminant hepatitis	Urgent liver transplantation (King’s College Criteria met)	Alive with liver transplant	Transplant
4	Gille N [46]	2020	France	Female	32	Immunocompetent	Fulminant hepatitis	Urgent liver transplantation (King’s College Criteria met)	Alive with liver transplant	Transplant
5	Sobala-Szczygieł B [83]	2016	Poland	Female	35	Immunosuppression	Acute liver failure	B19 in chronic HBV patient on pegylated interferon α-2a presenting with acute liver failure at week 23 of therapy	Resolution	3 months
6	Karrasch M [84]	2014	Germany	Male	27	Immunocompetent	Acute hepatitis	Concomitant acute primary EBV infection with associated hepatitis and probable B19 reactivation	Resolution	Improvement of general condition within 14 days
7	Bihari C [85]	2014	India	Male	19	Immunocompetent	Acute hepatitis	Diagnosis—suspected on bone marrow examination and diagnosed by IgM seropositivity	Resolution	N/R
8	González JM [86]	2012	Spain	Male	28	Immunocompetent	Acute hepatitis	Acute B19 hepatitis associated with arthralgias and myalgia	Resolution	8 months
9	Huang RJ [87]	2012	USA	Female	21	Immunocompetent	Fulminant hepatitis	Acute hepatitis that reached in the course of the disease AST of 9.348 U/L, ALT of 9.337 U/L	Resolution	2–3 weeks
10	Sun L [88]	2012	China	Female	34	Immunocompetent	Fulminant hepatitis	Acute B19 fulminant hepatitis associated with bone marrow failure	Resolution	3 months
11	Yang SH [40]	2011	Taiwan	Female	49	Under chemotherapy	Acute hepatitis	Rituximab-mediated reactivation of B19	Resolution	8 months
12	Hatakka A [89]	2011	Canada	Female	30	Immunocompetent	Acute hepatitis	Acute B19 hepatitis following a recent school outbreak of erythema infectiosum (‘slapped cheek disease’) attended by patient’s daughter	Resolution	Partial improvement within 9 days
13	Sun L [90]	2011	China	Male	33	Immunocompetent	Acute hepatitis	Clinical extrahepatic manifestation less severe	Resolution	2 months
14	Sun L [90]	2011	China	Female	50	Immunocompetent	Acute hepatitis	Clinical extrahepatic manifestation less severe	Resolution	2 months
15	Mogensen TH [91]	2010	Denmark	Female	50	Immunodeficiency	Chronic hepatitis	Chronic B19 hepatitis in patient with idiopathic lymphopenia, liver might have represented a viral reservoir, ALT normalization after B19 DNA clearance	Resolution	4 years
16	Krygier DS [92]	2009	Canada	Female	55	Immunocompetent	Fulminant hepatic failure	Fulminant B19 hepatic failure that required liver transplantation	Alive with liver transplant	Transplant
17	Lian QJ [93]	2009	China	Female	34	Immunocompetent	Acute hepatitis	B19 hepatic dysfunction associated with myelosuppression	Resolution	3 weeks
18	Pongratz G [42]	2009	Germany	Male	41	Immunocompetent	Acute hepatitis	Significantly increased frequencies of IFN-γ–producing CD4⁺ T cells specific to the VP1u region of B19—hypothesis that the intensity of liver involvement correlates with the likelihood of developing persistent B19 infection	Resolution	A few weeks
19	Ho JK [18]	2005	British Columbia	Female	34	Immunocompetent	Acute hepatitis	Severe and prolonged but self-limited acute B19 hepatitis, B19 DNA not detected in the serum	Resolution	10 weeks
20	Chehal A [94]	2002	Lebanon	Female	21	Immunocompetent	Acute hepatitis	Acute B19 hepatitis associated with acute HAV, complicated by pure red cell aplasia and autoimmune hemolytic anemia	Resolution	A couple of weeks
21	Hayakawa F [95]	2002	Japan	Female	19	Immunocompetent	Fulminant hepatitis	B19 transmission from Iv Ig; fulminant hepatitis by B19 infection following pre-existing coxsackie B4 infection	Resolution	1 month
22	Alliot C [96]	2001	France	Male	34	HIV	Acute hepatitis	B19 acute hepatitis associated with bone marrow failure; possible false-positive HBV test caused by B19	Death 18 days after admission	Death
23	Díaz F [43]	2000	Spain	Female	21	Immunocompetent	Acute hepatitis	Less severe hepatic involvement	Resolution	2 months
24	Díaz F [43]	2000	Spain	Female	43	Immunocompetent	Acute hepatitis	Less severe hepatic involvement	Resolution	3 weeks
25	Drago F [97]	1999	Italy	Female	41	Immunocompetent	Hepatic dysfunction	B19 acute hepatitis with purpuric exanthem. Serum B19 DNA probably negative because of a short viremic phase	Resolution	1 month
26	Hillingsø JG [72]	1998	Denmark	Female	41	Immunocompetent	Hepatic dysfunction	Hepatic dysfunction revealed after a recent B19 epidemic	Resolution	N/R
27	Hillingsø JG [72]	1998	Denmark	Female	35	Immunocompetent	Hepatic dysfunction	Hepatic dysfunction revealed after a recent B19 epidemic	Resolution	3 weeks
28	Longo G [68]	1998	Italy	Male	19	Immunocompetent	Acute hepatitis	Acute B19 hepatitis associated with Still’s disease and severe thrombocytopenia	Resolution	A couple of weeks
29	Pardi DS [98]	1998	USA	Female	58	Immunocompetent	Acute hepatitis	B19 acute hepatitis associated with aplastic anemia in patient with autoimmune hepatitis	Resolution	1 year
30	Pardi DS [98]	1998	USA	Female	47	Immunocompetent	Acute hepatitis	B19 acute hepatitis associated with pleuritis, mild interstitial pneumonitis, peribronchial lymphoid aggregates	Resolution	N/R
31	Nikkari S [99]	1996	Finland	Male	33	Immunocompetent	Acute hepatitis	Positive B19 DNA in the skin and serum. Acute B19 hepatitis associated with pleuropneumonitis, pericardial effusion	Resolution	2 months
32	Weinberg JM [100]	1996	USA	Male	18	Immunocompetent	Acute hepatitis	Acute B19 hepatitis associated with arthralgias and rash	Resolution	5 weeks

*ALT* alanine aminotransferase, *AST* aspartate aminotransferase, *AH* alcohol-related hepatitis, *B19* Parvovirus B19, *CD* + specific cluster of differentiation 4 positive, *DNA* deoxyribonucleic acid, *HAV* hepatitis A virus, *HBV* hepatitis B virus, *HIV* human immunodeficiency virus, *IFN-γ* interferon-gamma, *Ig* immunoglobulin, *IgG* immunoglobulin G*, IgM* immunoglobulin M, *Iv* intravenous, *MODS* multiple organ dysfunction syndrome, *N/R* not reported, *VP1u* VP1 unique region

Among the 28 patients reported as immunocompetent (87%), a predisposing factor or coexisting condition supporting the development of more significant liver damage was identified in 15 cases (53%), as detailed in Table 2.

**Table 2:** Overview of epidemiological features and associated risk factors in the 28 immunocompetent cases

Exposure to infection
Household/family exposure (mild respiratory symptoms) [87] Close contact with young primary school children (erythema infectiosum outbreak/common cold symptoms) [82, 89] Recent history of traveling (erythema infectiosum outbreak) [89, 100] Recent local B19 epidemic [72] Airplane passenger who assisted a visibly jaundiced individual [92] Vacation (group with common cold/diarrhea/erythema) [42] Recent transfusion with B19 contaminated blood products Red blood cell transfusion [48] Intravenous immune globulin transfusion [95]
Concomitant viral infections
Primary Epstein–Barr virus (EBV) with high EBV-DNA load [84] Concomitant acute hepatitis A [94] Concomitant acute hepatitis B (possible false positive test due to B19) [96] Pre-existing coxsackie B4 infection [95]
Autoimmune/immune-related/hematologic conditions
Autoimmune hepatitis confirmed [98], or suspected [46] Beta-Thalassemia trait [94]/minor [85]
Substance-related/drug-induced
Alcohol-related hepatitis, long-term drinking [48] Paracetamol (3 g/day for 7 days) [46] Recent treatment with valganciclovir [89]

Liver involvement varied considerably among the 32 reported cases. The spectrum ranged from mild hepatic dysfunction [72, 97] to acute liver failure and acute-on-chronic liver disease [91], as presented in Table S1. In three cases, the severity necessitated urgent liver transplantation [46, 92], highlighting the potentially life-threatening nature of the condition. Although liver biopsy was conducted in only a subset of patients, it offered valuable diagnostic insights when performed. Supplementary Table S2 provides detailed information on the liver biopsy and bone marrow biopsy findings, as well as the therapeutic approaches.

**Fig. 1:**
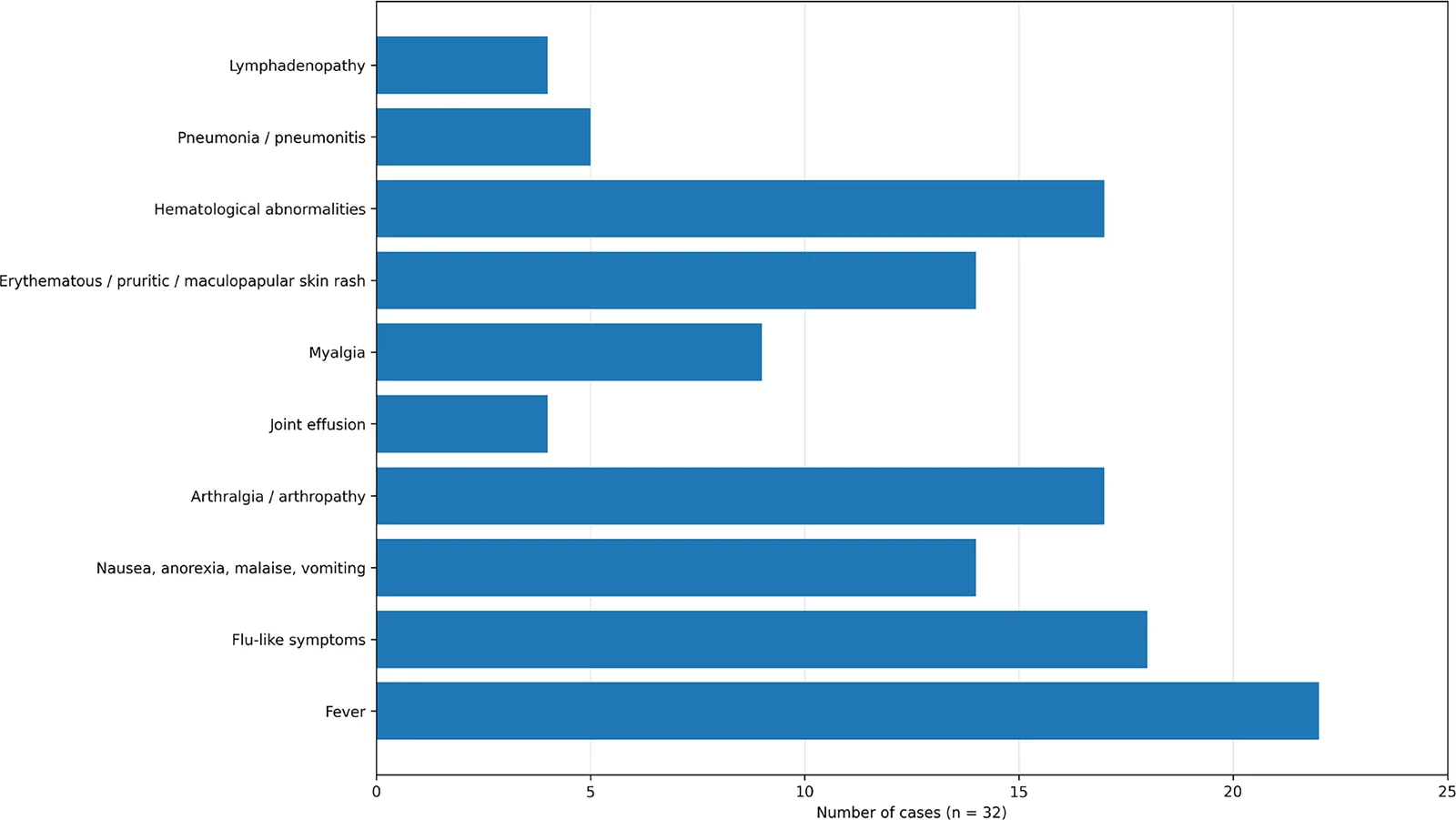
Frequency of extrahepatic symptoms, signs, and associated conditions reported among 32 published cases of parvovirus B19-related hepatitis. Values indicate the number of cases in which each manifestation was reported

Figure 1 illustrates the most commonly reported extrahepatic symptoms, signs, and associated conditions in the 32 cases of B19-associated hepatitis.

### Illustrative clinical case: acute cholestatic hepatitis revealing heterozygous ABCB11 deficiency

A 46-year-old immunocompetent Italian man presented to our emergency department with a 10-day history of progressively worsening systemic symptoms. Initially experiencing musculoskeletal pain managed with low doses of paracetamol, he subsequently developed fever (38–39 °C), sore throat, nausea, abdominal fullness, pruritus, and scleral jaundice, prompting medical evaluation.

The patient’s medical history was unremarkable, except for a previous diagnosis of Gilbert syndrome. Body weight before illness was 74 kg (h 177 cm, BMI 23.6 kg/m^2^). The patient had no previous liver-related events, was not receiving chronic medication, and had no history of perinatal abnormalities (including neonatal jaundice). On admission, excoriated lesions were observed on the thighs, likely due to scratching. The patient had no relevant risk factors for liver disease, including use of hepatotoxic agents or viral exposure. He reported minimal alcohol consumption and had no history of smoking.

**Fig. 2:**
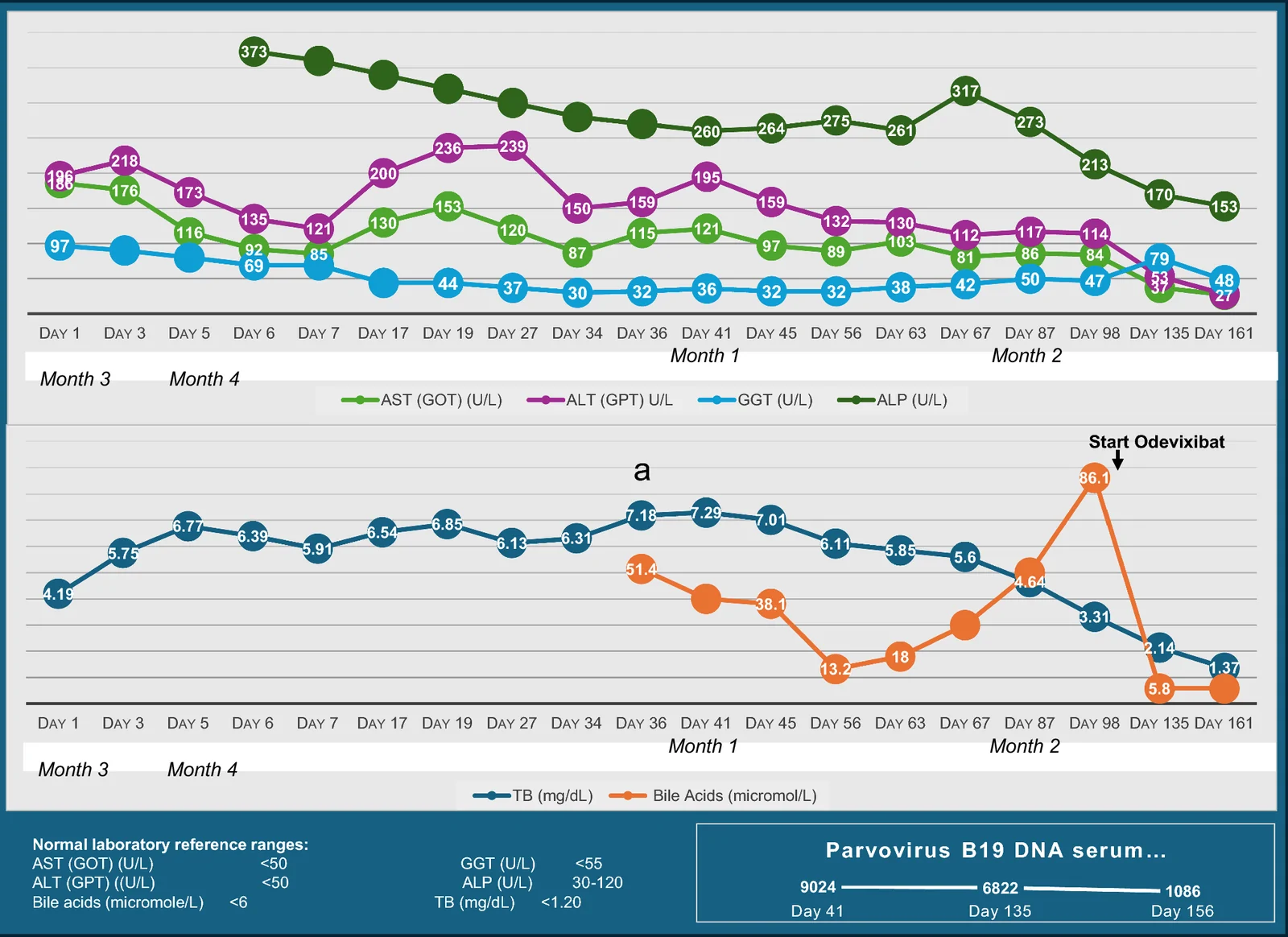
Temporal evolution of laboratory parameters. *ALP* alkaline phosphatase, *ALT* alanin aminotransferase, *AST* aspartate aminotransferase, *B19* parvovirus B19, *c/ml* copies per milliliter, *DNA* deoxyribonucleic acid, *GGT* gamma-glutamyl transpeptidase, *L* liter, *NGS* next generation sequencing, *Plt* platelets, *TB* total bilirubin, *U/L* International units per liter

Initial laboratory tests demonstrated a cholestatic-hepatocellular pattern of liver injury, characterized by elevated bilirubin and mild transaminase elevation. The detailed laboratory trends are reported in Table 3 and Fig. 2.

**Table 3:** Laboratory trends: case report

	WBC (10^9/L)	Neu (10^9/L)	Linfo (10^9/L)	Mono (10^9/L)	Hb (g/dL)	RG (10^12/L)	Plt (10^9/L)	INR	TB (mg/dL)	DB (mg/dL)	IB (mg/dL)	AST (U/L)	ALT (U/L)	GGT (U/L)	ALP (U/L)	Bile Acids (µmol/L)	CRP (mg/dl)
*Normal laboratory values*	*3.60–10.50*	*1.50–7.70*	*1.10–4.00*	*0.1–0.90*	*13.5–17.2*	*4.30–5.75*	*160–370*	< *1.20*	< *1.20*	< *0.30*	< *0.90*	< *50*	< *50*	< *55*	*30–120*	< *6*	< *0.50*
Day 1	11.94	1.92	9.3	0.63	13.3	4.26	183		4.19	2.51	1.68	186	196	97			0.51
Day 3	10.73	1.85	8.03	0.66	13.4	4.38	226	1.02	5.75	3.3	2.45	176	218				0.33
Day 5	8.2	1.67	5.79	0.6	13.6	4.42	261	0.99	6.77	3.58	3.19	116	173				0.3
Day 6	8.56	1.62	5.99	0.78	12.7	4.29	290		6.39	3.34	3.05	92	135	69	373		0.25
Day 7	8.37	1.79	5.79	0.66	12.8	4.05	303	1.02	5.91	3.01	2.9	85	121				0.17
Day 17	4.69	1.85	2.16	0.5	13.5	4.41	328		6.54	3.2	3.34	130	200	44			0.03
Day 19	4.26	1.7	1.87	0.54	13	4.28	273	0.98	6.85	3.17	3.68	153	236				0.02
Day 27	5.16	2.03	2.39	0.49	13.5	4.41	267		6.13	3.08	3.05	120	239	37			0.02
Day 34	4.65	1.92	2.01	0.44	12.7	4.28	244		6.31	3.04	3.27	87	150	30			0.02
Day 36									7.18	3.32	3.86	115	159	32		51.4	
Day 41	5.08	2.74	1.68	0.38	13.2	4.33	290	0.93	7.29	3.46	3.83	121	195	36	260		0.03
Day 45	6.08	3.66	1.75	0.44	12.5	4.06	319	0.96	7.01	3.26	3.75	97	159	32	264	38.1	0.02
Day 56	4.39	2.38	1.4	0.39	11.9	3.97	281	1.07	6.11	2.94	3.17	89	132	32	275	13.2	
Day 63	5.57	3.5	1.46	0.4	12	3.89	272	1.07	5.85	2.68	3.17	103	130	38	261	18	
Day 67	4.19	2.38	1.28	0.36	12.3	3.99	279	1.02	5.6	2.64	2.96	81	112	42	317		
Day 87	11.14	8.49	1.77	0.63	12.4	4.04	295	1.01	4.64	2.1	2.54	86	117	50	273		
Day 98	4.5	2.17	1.64	0.41	11.7	3.74	255	0.96	3.31	1.54	1.77	84	114	47	213	86.1	
Day 135	7.51	5.3	1.46	0.65	12.8	3.98	246	1.12	2.14	0.59	1.55	37	53	79	170	5.8	
Day 161	4.73	2.28	1.85	0.38	13.3	4.19	205	1.01	1.37	0.27	1.1	27	28	48	153		0.06

*ALP* alkaline phosphatase, *ALT* alanine aminotransferase, *AST* aspartate aminotransferase, *B19* parvovirus B19, *CRP* C-reactive protein, *DB* direct bilirubin, *DNA* deoxyribonucleic acid, *EBV* Epstein–Barr virus, *GGT* gamma-glutamyl transpeptidase,* g* grams, *Hb* hemoglobin, *IB* indirect bilirubin, *INR* international normalized ratio,* L* liter, *Lymph* lymphocytes, *mg* milligrams, *Mono* monocytes, *Neu* neutrophils, *Plt* platelets, *RBC* red blood cells, *TB* total bilirubin, *U/L* international units per liter, *WBC* white blood cells, *µmol/L* micromoles per liter

An abdominal ultrasound (US) showed a mildly enlarged liver (right lobe 16 cm) with regular margins and a homogeneous echotexture, as well as a mildly enlarged spleen (13.1 cm bipolar diameter; sectional area 62 cm^2^). The chest X-ray and echocardiogram were unremarkable.

Given the clinical presentation, a viral etiology of hepatitis was initially suspected. However, serologic testing for hepatitis A–D, cytomegalovirus (CMV), herpes simplex virus (HSV), and human immunodeficiency virus (HIV) was negative. The autoimmune and metabolic workup—including antinuclear antibody (ANA), anti-smooth muscle antibody (ASMA), antimitochondrial antibody (AMA), hemochromatosis and Wilson’s disease screening—was also unremarkable.

On day 7 of hospitalization, borderline EBV IgM positivity with positive IgG and mild DNA load (1409 copies/mL) initially led to a presumptive diagnosis of EBV-related hepatitis. However, the persistence of severe cholestasis, progressive weight loss (− 6 kg), and worsening clinical course prompted further investigations.

Serological and virological investigations for B19 revealed both IgM and IgG positivity, with a viral DNA load of 9024 copies/mL on day 41 of illness, consistent with an acute infection. Serum bile acids (BA) were 51.4 µmol/L (normal < 6 µmol/L) on day 36 of illness, with the patient still complaining of severe pruritus (Numerical Rating Scale 8/10) [101].

Pruritus caused sleep disturbance and scratching despite treatment with ursodeoxycholic acid (UDCA) and antihistaminic drugs. However, the prolonged liver failure, cholestasis, and the lack of clinical improvement, with only mild transaminase elevation and normalization of GGT (Table 3), raised suspicion of an underlying hepatic predisposition or associated liver disorder.

Vibration-controlled transient elastography (VCTE) showed a liver stiffness of 9.5 kPa (IQR/median = 5), theoretically suggesting advanced fibrosis (F3 according to the METAVIR scoring system). The controlled attenuation parameter (CAP) excluded the presence of steatosis (178 dB/m, SD 8), and the spleen stiffness did not suggest clinically significant portal hypertension (14.1 kPa, IQR 0.9).

The patient therefore underwent liver biopsy on day 76 of illness and urgent genetic testing for familial intrahepatic cholestasis in the attempt to clarify the clinical picture that was still characterized by severe cholestasis.

**Fig. 3:**
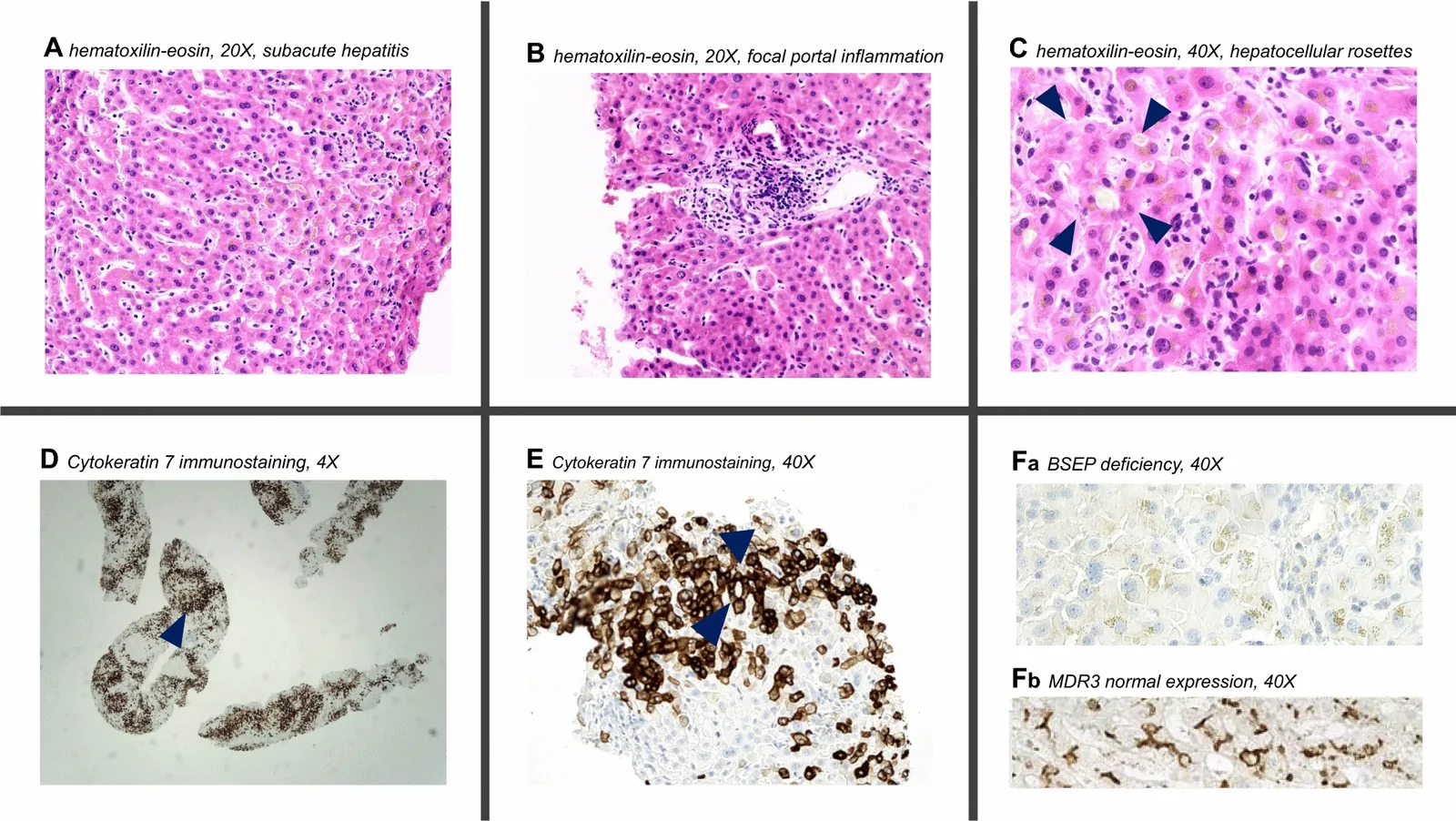
Histology findings. Legend: **C** arrows, hepatocellular rosettes, **D** arrow, aberrant cytokeratin 7 expression, **E** arrows, magnification of diffuse ductular metaplasia of hepatocytes highlighting the presence of rosettes, **Fa** marked reduction in BSEP (bile salt export pump) immunoreactivity, **Fb** multidrug resistance protein 3 (MDR3) normal immunoreactivity

Histology revealed subacute hepatitis with lobular necro-inflammatory foci (Fig. 3A), mild portal fibrosis and inflammation without features of significant interface hepatitis (Fig. 3B). Additionally there were hepatocellular cholestasis, hepatocellular rosettes and bile plugs (Fig. 3C). Macrovesicular steatosis was absent. Diffuse ductular metaplasia of hepatocytes was present, as shown by positive cytokeratin 7 (CK7) staining (Fig. 3D, E), with no evidence of ductular proliferation in zone 1. Fibrosis staging was 0 according to both Ishak and METAVIR scores. B19 DNA was detected in liver tissue by polymerase chain reaction (PCR) (2500 copies/microgram of DNA), confirming the diagnosis of B19-associated hepatitis. Immunohistochemistry was negative for EBV, CMV, and the expression of multidrug resistance protein 3 (MDR3). The expression of MDR3, related to ABCB4, the gene responsible for PFIC3, was normal. Notably, there was a marked loss of bile salt export pump (BSEP immunoreactivity) (Fig. 3Fa), supporting the diagnosis of an underlying transporter defect. Figure 3Fb shows preserved immunoreactivity of MDR3.

Given the severe cholestatic features and the significant reduction of BSEP expression on liver biopsy, next-generation sequencing (NGS), including 36 genes responsible for inherited liver disorders, was performed to further investigate the potential genetic cause of the cholestasis, using the Ion AmpliSeq NGS panel (Thermo Fisher Scientific Inc.). Genetic variants were classified according to the ACMG (American College of Medical Genetics and Genomics) guidelines [102] and the findings were subsequently confirmed by Sanger sequencing. The test revealed a heterozygous pathogenic variant in exon 16 of ABCB11 gene (NM_003742.4): c.1873dup p.Thr625Asnfs*6. This was consistent with BSEP deficiency.

Initial management of the patient included supportive care and treatment with high doses of UDCA (15–20 mg/kg/day), cholestyramine, and vitamin D supplementation (2000 IU/day), with no benefit.

Following genetic confirmation and in consideration of the elevated BA levels in the serum (Table 3, Fig. 2), and because of the persistence of disturbing pruritus (as only decreased from 8 to 5 on a NRS scale) [101], leading to scratching, sleep disturbance, and a further significant weight loss (− 3 kg), the patient was evaluated for therapy with odevixibat, an ileal bile acid transporter (IBAT) inhibitor.

The patient was treated with odevixibat at a dose of 40 µg/kg/day administered as a single morning dose, while UDCA therapy was continued for its potential synergistic effect, according to the prescribing information. A rapid clinical response was observed, with complete resolution of pruritus after 1 week of treatment, and almost complete resolution of jaundice within 2 weeks. Serial liver function tests demonstrated progressive biochemical improvement, with near-complete normalization after approximately two months of therapy, including normalization of serum BA levels (5.8 µmol/L; reference value < 6.0 µmol/L) and stabilization of total bilirubin at 2.14 mg/dL (direct bilirubin 0.59 mg/dL and indirect bilirubin 1.55 mg/dL). The patient also reported marked improvement in overall well-being, including progressive weight recovery (72 kg compared to a previous weight of 74 kg), lack of fatigue, and improved muscle tone. The total duration of odevixibat therapy was 12 weeks, during which no adverse effects were reported. Full laboratory trends during follow-up are presented in Table 3 and Fig. 2.

No recurrent episodes of cholestasis were observed during the available 1-year follow-up. Considering the underlying genetic condition, annual abdominal ultrasound for hepatocellular carcinoma (HCC) surveillance was recommended [103]. In addition, close monitoring for potential viral triggers was advised, along with avoidance or cautious use of medications known to precipitate cholestatic episodes, including amoxicillin, nonsteroidal anti-inflammatory drugs (NSAIDs), neuroleptic agents, and certain phytotherapeutic products.

Our report describes a case of subacute cholestatic hepatitis triggered by B19 infection, in which the clinical course was predominantly driven by an underlying, previously unrecognized ABCB11 deficiency. We have also shown how a brief course of IBAT inhibitor (odevixibat) therapy could markedly accelerate recovery in such cases.

## Discussion

### What does the present case and the review of the literature teach us?

The present work underlines the existence of parvovirus B19-associated hepatitis, in order to prevent under-diagnosis due to limited clinician awareness and insufficient routine testing. This narrative review demonstrates that primary B19 infection has a broad clinical spectrum of manifestations in adults, ranging from mild hepatic dysfunction to fulminant hepatic failure requiring liver transplantation, in which host factors and comorbidities play a significant role. In general, clinicians should take into consideration B19 infection in adult patients presenting with non-specific symptoms such as joint pain, skin rash, and flu-like illness.

The precise mechanisms of parvovirus B19 hepatic injury remain to be clarified, but evidence points toward a higher likelihood of clinical manifestations in individuals with predisposing conditions such as underlying hematologic disorders, coexisting liver disease, or possible genetic susceptibility. This is why critical judgment should always be in place and be deep, in order to understand whether the global clinical picture and the disease course are consistent with everything that is known at each time. In case of clashing information, additional comorbidities must be actively searched as well as other etiological factors. During the course of B19 infection, the avoidance of other hepatotoxic agents such as alcohol or acetaminophen may also be advisable to minimize additional liver injury.

Our case report highlights the value of modern personalized medicine, including genetic testing and serum BA dosing, in identifying underlying vulnerabilities in patients who are not immunocompromised. In such individuals, a genetic predisposition to inherited cholestatic diseases may play a role in the severity of hepatic involvement.

In this case, the early course was not consistent with an EBV-associated hepatitis. Specifically, the cholestatic pattern was too severe and persistent and not supported by the only mildly elevated liver enzymes; moreover, the EBV viral load was too low to justify liver failure. This prompted additional testing that led to the discovery of a recent B19 infection. However, despite the fact that the inflammatory pattern and the early generalized symptoms were indeed consistent with the B19 infection, the prolonged and symptomatic cholestatic pattern was not, leading the clinician to suspect and confirm a predisposing genetic cholestatic condition.

Furthermore, the latest EASL clinical practice guidelines on genetic cholestatic diseases recommend genetic testing for adults with unexplained liver conditions, with unusual features of a previously diagnosed liver disorder, or if they do not respond to initial treatment [104]. At the same time, it is advised to investigate environmental factors, such as infections, drugs, herbal remedies, and hormones, as potential triggers for a genetic cholestatic disease [104]. Among the genes responsible for PFIC, ABCB11 is the one most frequently associated in the heterozygous state with acute liver disease presentations, such as DILI, RIC, or hepatitis with an identified cause but an atypical course or presentation. This presentation, when associated with pruritus, low GGT levels, and elevated bile acids and alkaline phosphatase, should raise suspicion of BSEP (ABCB11) deficiency [104]. When BSEP disease and viral infections co-occur, the described mechanisms of liver injury include increased systemic inflammation. Because viral infections spike systemic inflammatory cytokines, notably IL-1β, they suppress the gene expression of BSEP. Consequently, when BSEP is already genetically compromised, this virus-induced suppression completely halts BA secretion, leading to a rapid and toxic accumulation of BA within the liver [105].

This approach led to a specific diagnosis that explained the persisting cholestatic pattern, allowing for the use of a new pharmacological approach, consisting of a short course (12 weeks) of an IBAT inhibitor (odevixibat). The treatment immediately resolved the difficult-to-treat pruritus and improved the quality of life in terms of sleep disturbances and social isolation [106].

A similar time-limited treatment and real-life strategy with IBAT inhibitors for episodic intrahepatic cholestasis was described in PFIC1 patients [106].

BSEP deficiency exhibits a genotype-dependent spectrum of disease severity, ranging from milder phenotypes with residual transporter activity (BSEP1), intermediate phenotypes (BSEP2), to severe forms associated with complete loss of BSEP function (BSEP3) [103]. This classification may have important therapeutic implications, as the presence of residual BSEP activity is associated with a greater likelihood of response to IBAT inhibitors and improved disease prognosis [107].

Odevixibat is an IBAT inhibitor indicated for the treatment of PFIC in the European Union, for the treatment of pruritus in PFIC in the United States [108], and for the treatment of cholestatic pruritus in Alagille syndrome in the European Union and the United States [109–111]. Patients with biallelic predicted protein truncating variants in ABCB11 gene exhibit a complete absence or lack of BSEP function and should not be treated due to lack of efficacy. In this case, liver histology demonstrated a partial rather than complete loss of BSEP expression, supporting the use of odevixibat [111].

## Conclusions

Although parvovirus B19 is a rare cause of acute liver injury, particularly in immunocompetent individuals, it should be considered in the differential diagnosis of unexplained hepatitis or acute liver dysfunction. This consideration is supported by the 32 cases reported in the literature and the additional case presented herein. B19 can act as a precipitating factor for acute liver dysfunction in genetically susceptible individuals, even without immunosuppression. The case report highlights the importance of investigating underlying genetic predispositions, such as BSEP deficiency, in cases of exceedingly severe or persistent cholestasis. To the best of our knowledge, this is the first case described in the literature where an IBAT inhibitor was successfully used in an adult patient with acute cholestasis due to B19 infection, exacerbated by a latent BSEP deficiency disease.

## Supplementary Information

Below is the link to the electronic supplementary material.

Supplementary file1 (DOCX 63 KB)

## Data Availability

Not applicable.
