# Supplementary material for: Parvovirus B19 infection as a trigger of acute cholestasis in heterozygous genetic BSEP deficiency: a case report and review of the literature of acute parvovirus B19-related hepatitis
Source: Intern Emerg Med. 2026 Jul 24;21(8):2799–816. doi: 10.1007/s11739-026-04464-1 (PMC13751806; doi:10.1007/s11739-026-04464-1)
Supplement: Supplementary file 1 [file 11739_2026_4464_MOESM1_ESM.docx]

Table S1. Overview of Laboratory Characteristics in 32 Adult Cases of Parvovirus-Related Hepatitis

Table S2. Other Characteristics in 32 Adult Cases of Parvovirus-Related Hepatitis
